# Function follows form: how the structure of neurons determines cortical network activity: Comment on: Hodapp A, Kaiser ME, Thome C, Ding L, Rozov A, Klumpp M, Stevens N, Stingl M, Sackmann T, Lehmann N, Draguhn A, Burgalossi A, Engelhardt M, Both M (2022) Dendritic axon origin enables information gating by perisomatic inhibition in pyramidal neurons. Science 377:1448-1452

**DOI:** 10.1007/s00424-022-02776-7

**Published:** 2022-12-03

**Authors:** Andreas Draguhn, Martin Both

**Affiliations:** Institute for Physiology and Pathophysiology, Medical Faculty, Heidelberg University, Im Neuenheimer Feld 326, 69120 Heidelberg, Germany

Textbooks provide a relatively simple, integrated view of the structure and function of neurons: synaptic input arrives at the richly branched dendrites, the resulting membrane potential fluctuations propagate passively to the soma and reach the axon initial segment. At this site, action potentials are generated whenever the potential crosses the voltage threshold for activation of sodium channels. In this scheme, neuronal computation is based on a linear integration of excitatory and inhibitory postsynaptic potentials which are translated into fast, all-or-none output signals.

The reductionist integrate-and-fire model of neurons has been quite useful to explain dynamic processes in networks. However, it has experienced important extensions over the years. We highlight two prominent examples: First, the complex form of neuronal dendrites can lead to strong attenuations of signals if they are generated at remote locations or small-diameter dendritic branches. Accordingly, high-resolution measurements have revealed several signal amplification mechanisms along dendrites, which may even give rise to dendritic spikes resembling action potentials [9]. Secondly, pyramidal neurons within cortical networks typically have a layered structure with distinct inputs arriving at different dendritic domains. A prominent recent hypothesis claims that activation of specific cortical networks goes along with a depolarization of the most distal dendrites by ‘unspecific’ excitatory synapses. The resulting depolarization amplifies more proximally inserting ‘specific’ inputs, e.g. from sensory neurons. The interplay between distal and proximal dendritic segments has been suggested to underlie directed attention or even consciousness [1]. Thus, dendritic structure and function allow for complex computations at the single-neuron level.

While most studies of neuronal signal integration focus on dendrites [8], more recent work points towards the importance of the axon’s location and properties [6]. Hodapp and coworkers 4 have now reported a new computational mechanism in cortical networks which is based on the location of the axon. Previous work by the same group had shown that in up to 50% of mouse hippocampal pyramidal cells, the axon emanates from a basal dendrite, rather than from the soma [10]. Functional analyses and computer modelling pointed towards a privileged function of the axon-carrying dendrite (AcD) in generating action potentials. Obviously, excitatory postsynaptic potential in AcDs reaches the axon initial segment without passing through the soma. This makes a major difference in situations of strong perisomatic inhibition, where inputs from other dendrites are attenuated or completely shunted by the strong, basket-like net of GABAergic synapses around the soma (Fig. 1). This notion constitutes an immediate link between the site of axon origin and the functional state of hippocampal networks: the hippocampus generates different patterns of coherent network oscillations which are largely organised by rhythmic activity of specific inhibitory interneurons [2, 5]. Hodapp and coworkers concentrated on a network state with particularly strong perisomatic inhibition, sharp wave-ripple complexes [3]. They asked whether neurons with dendritic axon origin are privileged to fire action potentials during this activity pattern. For this, they measured the activity of single hippocampal neurons together with network-level field recordings in awake, behaving mice. The juxtacellular recording technique [7] allowed subsequent staining and morphological analysis of the recorded cells. Thus, they could tell whether a recorded cell participated in SPW-R activity and whether the axon originated at the soma or at a basal dendrite. In line with their hypothesis, they found a massive preponderance of pyramidal cells with axon-carrying dendrites. This result was confirmed in mouse brain slices in vitro where a more detailed biophysical analysis revealed that the bias towards AcD cells was caused by the differential effects of perisomatic inhibition on cells with somatic versus dendritic axon origin. Thus, a major portion of hippocampal pyramidal cells are largely shut off by perisomatic inhibition during the SPW-R-state. This finding has important consequences for information processing in hippocampal-neocortical networks: SPW-R are involved in the readout of previously acquired spatial or declarative information into the neocortex, the putative site of long-term memory formation. The switch towards AcD cells during sharp wave-ripples means that this readout function goes along with a strong narrowing of the participating neuron population.

**Fig. 1:**
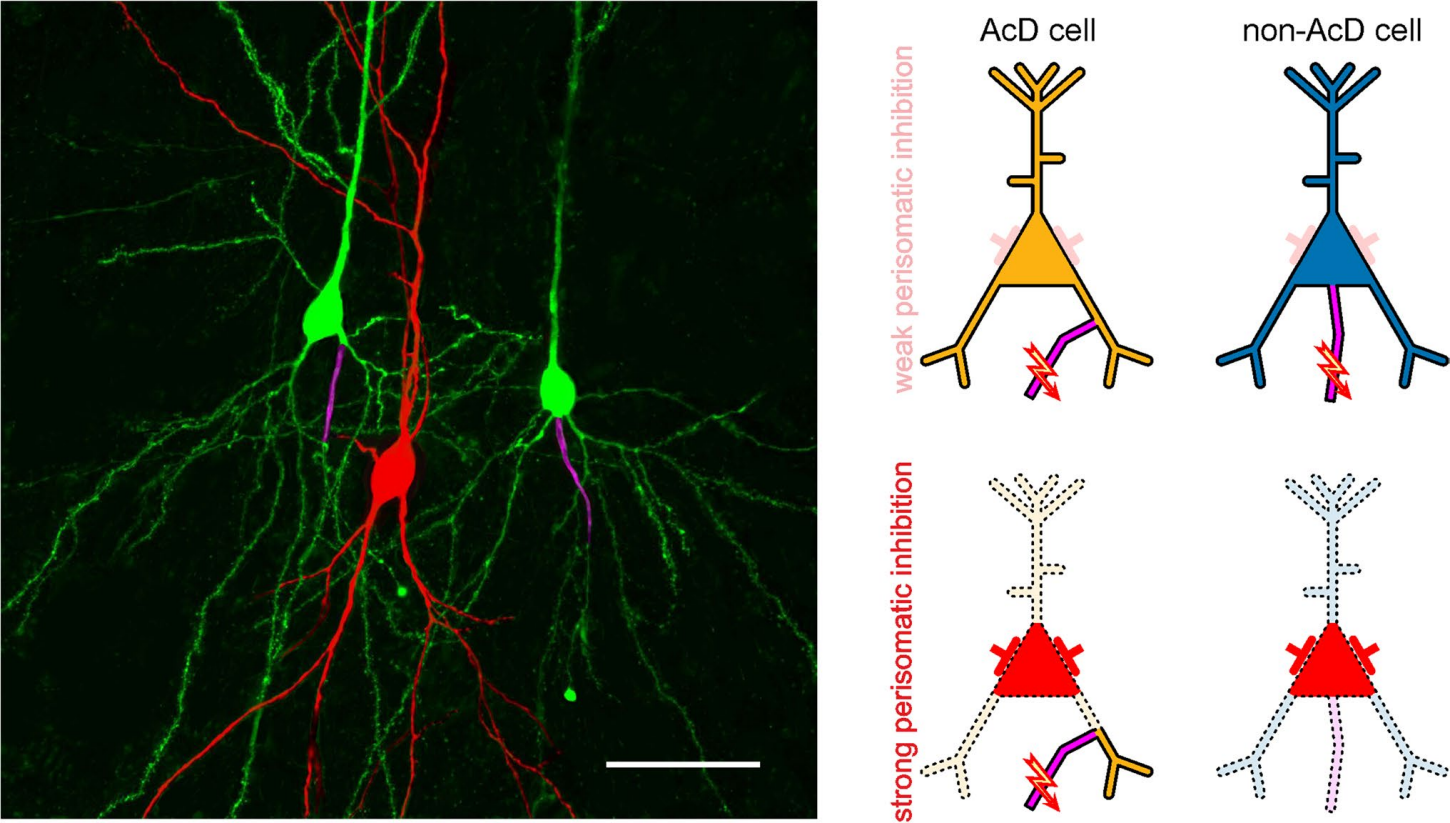
Left: reconstruction of hippocampal neurons after whole-cell recording, filling with biocytin and immunostaining against ß-IV-spectrin to mark the axon initial segment. AcD cell on the left (note that the axon emerges from a basal dendrite), non-AcD cell on the right (axon emerging from soma) and fast-spiking interneuron in the middle (highlighted in red). Right: schematic representation of action potential generation during weak (top panels) or strong perisomatic inhibition (bottom panels). During strong inhibition, the axon-carrying dendrite is the most likely source of excitation for action potential generation. Scale bar, 20 μm (adapted from [4]

The proposed mechanism is clearly relevant for understanding state-dependent, spatially distributed processes in neuronal networks. However, many questions remain presently open: is there experience-dependent plasticity of the axon origin? If so, what are the underlying mechanisms? Do AcD and non-AcD cells play different roles in further network states, e.g. during memory acquisition? Are cells within either population particularly strongly interconnected, forming distinct memory-encoding neuronal ensembles? Does the axon-carrying basal dendrite receive specific synaptic inputs? What is the function of AcD pyramidal cells in the neocortex, where they occur across species, including humans [11]?

It will take time to solve these and many related questions. Progress will depend on close cooperation between cellular and system neurophysiologists, cellular- and network-level neuroanatomists and theoretical neuroscientists. In this respect, the cooperative study conducted by Hodapp and coworkers [4] is an example for the increasing synergies between different approaches in modern neurosciences: cells and networks, function and form go together in nervous systems, and they must go together in our research.

## Data Availability

Not applicable.
